# Interaction between Calcium and Actin in Guard Cell and Pollen Signaling Networks

**DOI:** 10.3390/plants2040615

**Published:** 2013-10-15

**Authors:** Dong-Hua Chen, Biswa R. Acharya, Wei Liu, Wei Zhang

**Affiliations:** 1 The Key Laboratory of Plant Cell Engineering and Germplasm Innovation, Ministry of Education, College of Life Sciences, Shandong University, Jinan 250100, Shandong, China; E-Mail: donghua7103@sdu.edu.cn; 2 Biology Department, Penn State University, University Park, PA 16802, USA; E-Mail: bra2@psu.edu; 3 High-Tech Research Center, Shandong Academy of Agricultural Sciences, Key Laboratory of Genetic Improvement, Ecology and Physiology of Crops, Jinan 250100, Shandong, China; E-Mail: wheiliu@163.com

**Keywords:** actin, Ca^2+^ signal, ion channel, guard cell, pollen

## Abstract

Calcium (Ca^2+^) plays important roles in plant growth, development, and signal transduction. It is a vital nutrient for plant physical design, such as cell wall and membrane, and also serves as a counter-cation for biochemical, inorganic, and organic anions, and more particularly, its concentration change in cytosol is a ubiquitous second messenger in plant physiological signaling in responses to developmental and environmental stimuli. Actin cytoskeleton is well known for its importance in cellular architecture maintenance and its significance in cytoplasmic streaming and cell division. In plant cell system, the actin dynamics is a process of polymerization and de-polymerization of globular actin and filamentous actin and that acts as an active regulator for calcium signaling by controlling calcium evoked physiological responses. The elucidation of the interaction between calcium and actin dynamics will be helpful for further investigation of plant cell signaling networks at molecular level. This review mainly focuses on the recent advances in understanding the interaction between the two aforementioned signaling components in two well-established model systems of plant, guard cell, and pollen.

## 1. Introduction

Higher plants are equipped with signaling systems that play essential roles to fulfill their life cycles. Plant signaling transduction can be divided into several steps: the perception of developmental and environmental stimuli, the transduction of cellular signal by complex networks, and the physiological and morphological responses. Numerous ions, molecules, genes, proteins, and physiological processes and biochemical reactions are involved in a typical signaling cascade in a cell (e.g., guard cell ABA signaling) [1]. Biochemical, molecular, and genetic experiments have supported a comprehensive network of plant signaling even for limited plant physiological process carried by specific cells, e.g., guard cell invoked stomatal movements for transpiration, or pollen germination, and tube growth during pollination [2,3]. This review focuses on the significance of the two important signaling components, Ca^2+^ and actin cytoskeleton, especially on the interaction between them, in guard cells and pollens.

Ca^2+^ is an indisputably important element for both cell structure and signaling during plant growth and development, and also the accumulating data demonstrate its indubitable significance in plant nutrition. Furthermore, recent researches support the concept of cytosolic free Ca^2+^ ([Ca^2+^]_cyt_) as an intracellular second messenger that mediates developmental and environmental stimuli into plant physiological and biochemical responses [4,5]. The investigations on the latter topic as how Ca^2+^ as one of the most important regulator in plant signaling have been carried out from whole-plant to single protein levels [5]. [Ca^2+^]_cyt_ is maintained lower than 100 nM in resting plant cells while Ca^2+^ in the extracellular and organellar levels is at millimolar level [5]. The elevation or oscillation of free Ca^2+^ concentration in the cytosol acts as the [Ca^2+^]_cyt_ signal, which is the result of stimulus triggered Ca^2+^ influx from extracellular and release from endo-organelles (e.g., vacuole and endoplasmic reticulum) through Ca^2+^-permeable channels on plasmamembrane and endomembrane, respectively [6,7,8,9]. Many research groups have been attempting to discover upstream components and downstream effectors of Ca^2+^ signaling network.

Actin dynamics play important roles in many cellular signaling cascades in plants. In almost all eukaryotic cells, actin exists in two forms: filamentous actin (F-actin) and monomeric actin or globular actin (G-actin). F-actin filaments (with 5 to 7 nm diameter) play important roles in cell shaping, division, growth, vesicle and organelle movements, cell death, as well as signaling in responses to biotic and abiotic stresses [10,11,12,13]. G-actin can polymerize into F-actin with a characteristic of architecture and polarity in cytosol and could form actin filament bundles or cables as actin cytoskeleton. The actin dynamics as polymerization and de-polymerization between the G-actin and F-actin is an essential process for actin function, and could be regulated by developmental and environmental signals [14]. The actin dynamics needs the existence of enough G-actin accompanies with visible F-actin bundles. Indeed, in Arabidopsis pollens, the G-actin is estimated at very high concentration to about 50 µM [15], and only very small amount of a plant cell’s G-actin polymerizes into F-actin [16]. The superabundance of unpolymerized G-actin in plant cells suggests they are readily available for quick polymerization in response of stimuli [17], and also indicates the highly dynamic characteristic of actin network.

## 2. Why Guard Cells and Pollens

Not only cells are the structural and functional unit of all organisms but also vital responder to internal and external stimuli. Hence, the study of signaling network at cellular level is very important. Plants contain many specialized cells, for example, guard cells and pollens. The characteristic of a specialized cell is controlled by expression of a particular set of genes, while keeping the others repressed, and epigenetic regulation is also involved for their differentiation processes during development. The difference between guard cells and pollens has been identified by large-scale transcriptomic and proteomic analyses and the hypothesis has been proved [18,19,20,21,22,23]. These findings indicate that the signaling pathways maybe different among the different plant cell types or even different developmental stages of same cell type. Consequently, the comparison the signaling network of different specialized plant cells and the cells at different stages of similar cell type will be very helpful for understanding the common signaling mechanisms in response to developmental and environmental stimuli.

Leaves are the main photosynthetic organs of plant and are important for plant vegetative growth. In addition, leaves are also important for transpiration, and play important roles in response to biotic and abiotic stresses to cope with the environment and plant survivability [24,25,26]. The absorption of atmospheric carbon dioxide (CO_2_) (a substrate for carbohydrate biosynthesis by photosynthetic reaction), and the loss of water vapor by transpiration, are regulated by stomata (the microscopic pores present on the leaf surfaces). Stomata are consisted of guard cells surrounded in pairs, and are separated functional complex with no plasmodesmata connected with the contacted epidermal cells. Stomata are also major entry points for pathogen invasion. The opening and closing of stoma are important for gas exchange as well as to cope with the environment and plant survivability in response to phytohormone and environmental stresses [27,28,29]. Many internal and external (biotic and abiotic) stimuli are well known regulators of stomatal aperture, including hormones (e.g., ABA, auxin, and ethylene), blue and red light, water status, CO_2_ concentration, circadian clock, temperature, and pathogen [2,30,31,32]. The stomatal movements are controlled by the volume change of guard cells, and the volume change is the result of turgor alteration. During stomatal opening, guard cells accumulate solutes such as potassium, anions, and malate, and the increased osmolality enables water uptake, swelling of guard cells that in turn causes opening of stomata. In the process of stomatal closing, guard cells lose osmotic solute by efflux mechanism, and malate metabolizes into osmotically inactive starch that in turn facilitates shrinking of guard cells and closing of stomata [2,31,32,33]. Because of their physiological importance, guard cells have been well studied at whole-plant, whole-leaf, cellular, subcellular, and molecular levels by using different investigation tools, and they have been developed as the first model system for plant cell signaling. Both, Ca^2+^ and actin dynamics, have been suggested to be involved in guard cell singling networks.

After the transition from vegetative growth into reproductive growth stage, the fertilization is the most important process for sexual reproduction in flowering plants, and that begins with landing of the mature pollen on the top of a suitable pistil, followed by pollen hydration, germination, and pollen tube polar growth, and fertilization of the female gametophyte [34]. During the processes of pollen germination and tube elongation, many factors, such as Ca^2+^, K^+^, H^+^, Cl^−^, NO, ROS, and cytoskeletal components, are involved in the regulatory network [34,35,36,37,38,39]. Among these regulatory factors, Ca^2+^ has been indicated as a crucial player in pollen signaling [4,40,41], and actin organization patterns and actin dynamics have been implicated in cellular signaling as well as in cellular architecture [11,42,43]. As pollens and pollen tubes are representatives for the investigation of haploid and polarized growth of plant cells, together with their significance in double fertilization, pollens and pollen tubes have been regarded as very important systems for reproduction and cellular signaling studies.

Both, guard cells and pollens, are well established model systems for investigation of plant signaling networks, so the similarities and differences between them will give us common signaling characteristics of plant cells, and the accordance of specific cell functioning and signaling. About 20 years ago, the elevation of [Ca^2+^]_cyt_ was shown to trigger the fragmentation of actin bundles in pollen tubes and actin reorganization in guard cells [44,45]. Research findings indicate that the disruption of actin also affects the physiological processes during pollen germination, pollen tube growth, and stomatal movements [34,43,46,47,48]. The actin dynamics also regulate Ca^2+^-permeable channel activity and [Ca^2+^]_cyt_ signal in pollens and guard cells [41,49,50]. These data suggest the existence of a complicated regulatory feedback loop between the two important signaling elements, [Ca^2+^]_cyt_ and actin.

## 3. Means to Study Ca^2+^ and Actin Signaling

Without the aid of developed microscopy, biochemical, and genetic techniques for plant cell investigation, it would be impossible to detect the changes in the levels of [Ca^2+^]_cyt_, the structure or/and dynamics of actin, and to determine the roles of Ca^2+^ and actin in diverse physiological and developmental processes in guard cell and pollen signaling.

Calcium imaging is the very powerful and visible technique to study the change of [Ca^2+^]_cyt_ in living plant cells. [Ca^2+^]_cyt_ concentration change shows complex patterns, such as spiking or oscillation in response to upstream regulators and, thus, to trigger the downstream cellular responses [5,7]. In response to developmental or environmental cues, [Ca^2+^]_cyt_ concentration can be varied from 100 nM (for maintenance of resting cells) to above μM level (during activated state of the cells). As high concentrations of [Ca^2+^]_cyt_ is toxic to living cells, the elevation of [Ca^2+^]_cyt_ is brief and/or subcellularly restricted within the physiological range. A set of small-molecules, Ca^2+^ sensing fluorescent dyes (e.g., Fluo-3, Fura-2) have been widely used to detect [Ca^2+^]_cyt_ changes in plant cells [41,50,51,52,53], and the advantage of these dyes is that they can easily be applied to all kinds of plant cells [52,54,55]. FRET-based Ca^2+^ sensors, such as yellow cameleon (YC) 2.1 and higher versions of yellow cameleon (e.g., YC 3.6), are considered as a more sensitive and efficient method than to fluorescent dyes [9,56]. To detect the relative or quantitative change of [Ca^2+^]_cyt_, a fluorescent microscope is needed for Ca^2+^ sensing fluorescent dye application, and FRET-based sensor methods require a confocal microscope.

It is well known that, Ca^2+^ channels located on the plasmamembrane and endomembrane facilitate the Ca^2+^ influx and [Ca^2+^]_cyt_ rise that, in turn, cause changes of [Ca^2+^]_cyt_. Based on above facts, these channels could be regarded as direct upstream elements for generation of [Ca^2+^]_cyt_ in plant cells. Patch clamp technique enables the recording of Ca^2+^ channels in plant cells [6,7,8,57]. Due to the poor selectivity of plant cell Ca^2+^ channels for the cations, a Ca^2+^ channel is referred as Ca^2+^-permeable channel [58,59,60]. By using electrophysiological means (the activation and gating mechanisms), several kinds of Ca^2+^-permeable channels have been recorded in the plasma membrane, tonoplast, endoplasmic reticulum, chloroplast, and nuclear membranes of plant cells, including depolarization-activated, hyperpolarization-activated, voltage-independent, stretch-activated, and cyclic nucleotide gated (CNG) Ca^2+^ channels, as well as their pharmacological characteristics [7,50,60,61,62]. In addition, the application of stretch-activated Ca^2+^ channel blocker, Gd^3+^, stops the osmotic stress induced [Ca^2+^]_cyt_ elevation, indicating the opening of plasma membrane Ca^2+^ channels is essential to initiate the [Ca^2+^]_cyt_ signaling [50]. Several gene families have been suggested as Ca^2+^ channel candidates, in Arabidopsis, that include cyclic nucleotide gated channel family (CNGC, 20 members), glutamate receptor homolog family (GLR, 20 members), and a two-pore channel (TPC1) [8,61,63,64,65].

To accomplish biochemical and physiological responses of the [Ca^2+^]_cyt_ signals triggered by internal or external stimuli, downstream Ca^2+^ sensors play vital roles in the perception and decoding of Ca^2+^ signals. There are four gene families with EF-hand Ca^2+^ binding domain that have been suggested as Ca^2+^ sensors in plants, and these families include calmodulin family, calmodulin-like protein (CML) family, Ca^2+^-dependent protein kinase (CDPK or CPK) family, and the calcineurin B-like protein (CBL) family [66,67]. The large number of calcium sensor proteins share different Ca^2+^-binding characteristics, and with diverse subcellular localizations and distinct downstream signaling interactions that enable these proteins to decode the information related to Ca^2+^ oscillations and spikes and subsequently to process of the information into appropriate cellular function.

To study the function and organization of actin during plant development and signaling, pharmacological agent(s) (drug) that causes disruption of actin filaments (e.g., latrunculin B), causes stabilization of actin filaments (e.g., phalloidin), and blocks actin polymerization (cytochalasin), were used previously [68,69,70,71]. Besides pharmacological agents, fluorescent methods were also usually used to visualize actin and were applied for actin imaging, prior to the discovery of fluorescent proteins [72]. Fluorescent proteins have the higher efficiency than fluorescent dyes to visualize actin organization and actin dynamics in living plant cells. GFP fused with actin binding domain or actin regulatory proteins have been demonstrated to bind F-actin *in vivo* [59,73,74,75]. Furthermore, a green to red photo-convertible probe, mEosFp::FABD-mTn, has been shown to work with higher precision for labeling F-actin and can be used for visualization of actin dynamics and interactions with microtubules and other organelles [76]. Lifeact-mEGFP was also suggested as a new versatile probe of F-actin without changing actin dynamics nor the physiological activities of living plant cells [77,78]. These actin labeling and imaging techniques supplied powerful tools for study of physiological roles of plant actin skeleton and actin dynamics in living cells.

## 4. Crosstalk of Actin and Ca^2+^ in Plant Cells

During developmental processes and in response to various stresses, most often, plant cells show both change of [Ca^2+^]_cyt_ and alteration of actin structure. For instance, during stomatal movements, Ca^2+^ signal activation is needed for both opening and closure, and rapid actin rearrangement is also essential for normal stomatal function [2,28,44,79,80]. The same phenomena have also been reported in other plant cells, such as root hairs during their growth [81,82], xylem cells during their differentiation [83,84,85], and in pollens during their germination and tube elongation [34,45]. Below, we elucidate the signaling crosstalk between Ca^2+^ and actin in guard cell and pollen systems.

### 4.1. Ca^2+^ and Actin in Guard Cells

In guard cell signaling networks, multiple stimuli that result in change of stomatal aperture mostly utilize [Ca^2+^]_cyt_ as a second messenger. In the well-established stomatal movement system, for ABA induced stomatal closing and ABA inhibited stomatal opening processes, [Ca^2+^]_cyt_ elevation or oscillation could usually be seen, even in Ca^2+^-independent signaling pathway (e.g., pH pathway) [2,31,86,87,88,89,90,91]. A recent report also indicates that [Ca^2+^]_cyt_ plays a positive regulatory role in ABA activation of pH_cyt_ [92]. [Ca^2+^]_cyt_ elevation has been shown to inactivate inward K^+^ channels and activate slow anion channels [87] and, thus, leading to stomatal closure [93]. In auxin, blue light and Cyclic GMP induced stomatal opening, [Ca^2+^]_cyt_ elevations have been observed [94,95,96].

In addition to ABA, other abiotic stimuli such as high CO_2_, cold shock, ROS, and biotic-stress by pathogens also cause [Ca^2+^]_cyt_ elevations and stomatal closure [57,97,98,99]. The removal of extracellular Ca^2+^ using EGTA abolishes [Ca^2+^]_cyt_ elevations in response to H_2_O_2_, cyclic GMP and hypo-osmolality, indicating plasma membrane Ca^2+^ influx is essential to initiate the [Ca^2+^]_cyt_-mediated signaling [50,57,100]. These reports suggest that the importance of Ca^2+^ influx through the Ca^2+^-permeable channels on the plasma membrane for generation of [Ca^2+^]_cyt_, and its role in stomatal movements, are response to both abiotic and biotic stresses.

Both CNGC1 and CNGC2, two members of CNGC family, are permeable to Ca^2+^, but also permeate K^+^ and are regulated by cyclic nucleotides, calmodulin, and pathogens. The activation of these CNGC channels in response to pathogen generates Ca^2+^ (Ba^2+^), current in Arabidopsis guard cell protoplasts, suggesting a possible role of CNG channels in Ca^2+^-based signaling in this cell type [101,102,103,104,105]. AtTPC1 channel also has been indicated to be involved in Ca^2+^ signaling cascades [106]. Recently, AtTPC1 has been shown to encode a vacuolar two pore channel 1 and its function has been implicated in Ca^2+^ signaling and stomatal closure [63,107,108].

In guard cell signaling several Ca^2+^ sensors have been identified that sense and amplify the Ca^2+^ signal. In *Arabidopsis thaliana*, CPK23 exhibits a stoma phenotype under water stress. T-DNA knockout mutant plants of *CPK23* gene show enhanced drought resistance and decrease stomatal aperture, while its overexpression lines show hypersensitivity to drought and wider stomatal aperture [109]; CPK3 and 6 are involved in ABA regulation of guard cell slow-type currents [110], CPK6 also is a positive regulator in methyl jasmonate signaling in guard cells [111]; CPK4 and CPK11 phosphorylate ABF4 and ABF1 (two members of the ABA-RESPONSIVE ELEMENT BINDING FACTORS (ABFs)) *in vitro*, and act as positive regulators of stomatal movements in calcium-mediated ABA signaling pathways [112]. SLAC1, a slow anion channel on the plasma membrane, is important for stomatal closing, could be activated by coexpression with CPK21 and CPK23 heterogeneously [113,114,115]. In addition to CPKs, other calcium sensors have been identified. CBL1 and CBL9, two members of CBLs, have been shown to play important roles on guard cell ABA signaling and interact with CIPK23. The *cbl1 cbl9* double mutant plants show hypersensitive to ABA regulation of stomatal movements and more resistance to drought treatment [116]. Extracellular application of calmodulin induces stomatal closure via a G-protein, nitric oxide, and H_2_O_2_ invoked pathway [117,118], suggesting the possible importance of calmodulin in regulation of guard cell signaling downstream of Ca^2+^ signal. CML9 and CML4 are involved in stress responses including ABA-inhibited seed germination and young seedling growth [119,120], however, the function of this family in guard cell Ca^2+^ signaling still needs further investigation.

Actin dynamics plays important role during stomatal movements. During light inducted stomatal opening and the dark or ABA induced stomatal closing, the actin reorganizes between a radial pattern (opening status) and a randomly oriented and short-fragmented pattern (closure status) [44,46], and the stomatal opening induced by fusicoccin is delayed with the pretreatment with phalloidin (a pharmacological agent that prevents de-polymerization of actin filaments) [70]. The ABA-insensitive mutant (*abi1-1*), which is impaired in ABA induced stomatal closure, fails to depolymerize actin and stomatal closure [121,122,123]. In wild type Arabidopsis plant, a small guanosine triphosphatase (GTPase) protein AtRac1 is inactivated by ABA, and the inactivation of AtRac1 GTPases induces disruption of the guard cell actin cytoskeleton and stomatal closure, while in *abi1-1*, neither AtRAC1 inactivation nor actin cytoskeleton disruption nor stomatal closure happens [124], indicating ABI1 functions upstream of AtRAC1 and actin in guard cell ABA signaling. ABA induced actin disruption could be dismissed by removal of external Ca^2+^ with application of EGTA, and treatment of Ca^2+^ could mimic the ABA induced actin rearrangement is guard cells, suggesting the Ca^2+^ regulation of actin dynamic pathway is important in ABA-mediated stomatal movements [70]. Osmotic stress is also involved in actin dynamics and stomatal movements [125]. In Arabidopsis, ARP2 (Actin Related Protein) encodes ARPC2 subunit of the ARP2/3 complex [126]. The *arp2* mutant plants are less sensitive to ABA and CaCl_2_-induced stomatal closure and do not show actin reorganization in response to ABA. Upon addition of cytochalasin D (actin modifying agent that induces de-polymerization of actin filaments) *arp2* mutant guard cells show similar ABA-mediated stomatal closure as wild type, indicating that ABA acts through ARP2/3 complex in ABA disruption or de-polymerization of actin [126]. Recently, a plant specific actin binding protein, SCAB1, has been identified and that interacts with F-actin and plays a regulatory role in ABA-mediated stomatal response [127]. These findings indicate the importance of actin dynamics in stomatal movements, but it appears that actin skeleton is rearranged passively.

Accumulating evidences show that actin is also an active regulator of stomatal movements. Cytochalasin D, which could depolymerize the F-actin, activates K^+^_in_ channels and enhances light-induced stomatal opening. Similarly, application of actin filament stabilizer, phalloidin, inhibits of K^+^_in_ currents and light-induced stomatal opening [80]. Cytochalasin D also restores the hyperosmolarity inhibition of K^+^_in_ currents [125], suggesting the depolymerized actin is a positive activator of K^+^_in_ channels during stomatal movements. More importantly, actin depolymeirzation induced by Cytochalasin D or hypo-tonic external solution, also activates guard cell plasma membrane localized Ca^2+^-permeable channels (both voltage-dependent and stretch-activated Ca^2+^-permeable channels) and currents at both single channel and whole-cell recording levels, suggesting the actin dynamics is downstream of osmotic stress but an upstream regulator of [Ca^2+^]_cyt_ signal [49,50]. Actin dynamics has also been shown to regulate both ion release from vacuole and Ca^2+^ entry mediated by plasma membrane localized Ca^2+^-permeable channels in guard cells during ABA induced stomatal closing [128]. Altogether, these findings indicate that both [Ca^2+^]_cyt_ and actin dynamics are regulated by a complex inter-regulatory loop in guard cells during stomatal movements.

### 4.2. Calcium and Actin in Pollens and Pollen Tubes

Ca^2+^ also plays important roles in pollen germination and in pollen tube growth, and the crucial effect of this ion during these processes has been described in the 1960s [129]. With the development of advanced biological techniques, now we know much more about the role of Ca^2+^ signaling during pollen germination and tube growth, which includes influx of Ca^2+^ and other ions in pollens, and the tip-focused Ca^2+^ gradient (in pollen tubes, the [Ca^2+^]_cyt_ ranges from 2–10 μM in the apical region, 20 μm at the tip, and 20–200 nm in the shank) and Ca^2+^ oscillations, apical Ca^2+^ entry and other osmotic ion influx through ion channels. As well, the contribution of Ca^2+^ in polar growth of the tube has been firmly established, and inhibition of external Ca^2+^ entry in pollen or pollen tube or disruption of Ca^2+^ gradient in the growing tube has been shown to prohibit the normal germination and elongation [8,41,130,131,132,133,134,135,136,137].

Entry of Ca^2+^ from outside for generation of [Ca^2+^]_cyt_ is important for pollen germination, as well as for pollen tube growth and orientation [40,129,131,137,138]. By using Ca^2+^-selective vibrating probes, pharmacological treatments, together with electrical fields and ionophoretic microinjection method and the comparison of [Ca^2+^]_cyt_ dynamic between non-growing and growing pollen tubes, it has been shown that Ca^2+^ channels (e.g., voltage-gated Ca^2+^ channels) in the plasma membrane of the pollen tubes are important for regulation of [Ca^2+^]_cyt_ and tube growth [132,139,140,141]. By using patch clamp technique, stress-activated Ca^2+^ channels have been identified in both lily pollens and pollen tubes [142], and voltage-dependent Ca^2+^-permeable channels of pollens also have been identified in different plant species [142,143,144,145]. These reports indicate that the activation of these channels is important for [Ca^2+^]_cyt_ generation [41]. Furthermore, genes encoding Ca^2+^-permeable channels have been identified in pollens. The CNGC18, a plasma membrane localized cyclic nucleotide-gated channel of Arabidopsis, is expressed at the tip of the pollen tube and is essential for normal tip growth. In addition, expression of CNGC18 has been shown to accumulate Ca^2+^ in *E. coli*, indicating that CNDC18 may be a Ca^2+^-permeable channel [146]. D-Serine activation of pollen Ca^2+^ currents, [Ca^2+^]_cyt_ generation and tube growth, together with the data from Ca^2+^-specific vibrating probe and physiological assays by using glutamate receptor like genes, indicating that GLRs are functional Ca^2+^-channels important for pollen tube elongation [38].

Downstream of [Ca^2+^]_cyt_ in pollen and pollen tube signaling, multiple Ca^2+^ sensors have been identified that transduce the [Ca^2+^]_cyt_ signals into physiological and biochemical responses. Growing pollen tubes exhibit higher activity of the CDPK kinase in the apical region, whereas nongrowing tubes show uniform kinase activity. Moreover, the modification of the kinase activity changes the orientation of the tubes, and the increased [Ca^2+^]_cyt_ enhances the kinase activity that, in turn, leads to reorientation of pollen tube [147]. Microinjection of fluorescence labelled calmodulin into pollen tubes indicated evenly distribution of calmodulin, but the higher calmodulin activity was observed in the apex, and that was correlative with [Ca^2+^]_cyt_-concentration distribution [147].These data suggest that CDPKs may play important roles in pollen germination and tube growth. In *Petunia*, overexpression of PM localized PiCDPK1 seriously affected the polarity of pollen-tube growth and [Ca^2+^]_cyt_ [148]. CDPK24 and 32, CBL2, and CBL3 were identified by a large-scale screening of Ca^2+^ sensors in pollen tubes and were found to be involved in regulating pollen-tube function [20]. CPK17 and CPK34 play important roles in Ca^2+^ signaling to increase the growth rate of pollen-tube tip [149]. These works open the door to access the complex signaling network downstream of [Ca^2+^]_cyt_. However, up to now, as researchers have limiting knowledge on overall pollen expressing Ca^2+^ sensors, we need to go a long way to clarify and to identify the downstream components and their interacting partners of Ca^2+^ signaling network in pollens and pollen tubes.

Actin cytoskeleton in pollens and pollen tubes has been emphasized since long time, and has been implicated in protoplasmic streaming and vesicle trafficking, which are essential for pollen-tube tip growth [150,151]. Pollen-tube actin filaments can be categorized into three distinct patterns: longitudinal actin cables found in the pollen tube shank; dense actin structures called actin fringes or collars at the subapex; and highly dynamic, but less abundant actin filaments found at the extreme distal pollen-tube tip [152,153]. Up to now, it has been found that the alteration of actin dynamics or change of actin pattern in living cells by cytochalasin B, phalloidin, profillin (monomeric actin-binding protein), actin-binding proteins (e.g., villins and myosins), and small G proteins, results in disruption of normal pollen germination and pollen-tube growth [15,37,39,59,69,71,72,150,151,153,154,155,156,157,158,159,160,161,162,163]. In addition, multiple reports indicate association between actin pattern and Ca^2+^ gradient [45,161,164]. F-actin co-localizes with CDPK, and CDPK does not interact with actin directly, and actin binding proteins such as 135-kilodalton actin-bundling protein and myosin XI could be regulated by [Ca^2+^]_cyt_ in regulation of actin bundling, suggesting that actin might be indirectly regulated by Ca^2+^ [165], these findings indicate that Ca^2+^ is an essential element in regulation of actin dynamics. On the contrary, actin also has been shown as a positive regulator of [Ca^2+^]_cyt_ in pollens. In the study by Wang *et al*., 2004, treatment of F-actin de-polymerization reagents, cytochalasin B and D, activate Ca^2+^-permeable channels in the plasma membrane of Arabidopsis pollen protoplasts, and also triggers [Ca^2+^]_cyt_ elevation [41]. In addition, the authors have shown that the activation of Ca^2+^-channels and [Ca^2+^]_cyt_ could be repressed by pretreatment of phalloidin, indicating that the regulation of Ca^2+^ influx through Ca^2+^-permeable channels is dependent on the actin dynamics and the de-polymerization status of actin is a positive regulator for generation of [Ca^2+^]_cyt_ [41].

## 5. Conclusions and Perspectives

During the development and physiological response to external stimuli processes, both [Ca^2+^]_cyt_ and actin network dynamics are important messengers of signaling in guard cells, pollens, and other plant cells. In this review, we briefly described individual roles of each mentioned messengers and compared the relationship of these two messengers in the two growing plant cell types: the reversible growing model cell, guard cell, and the tip polar growing model cell, pollen. Accumulating data show the existence of the inter-regulation of [Ca^2+^]_cyt_ and actin in these plant cells, and the importance of interaction between them for the normal function of these cells for the plant life. But the fundamental mechanism for the interaction between these two signaling components is still largely unknown, the [Ca^2+^]_cyt_ regulation of actin dynamics looks easier to understand, which is primarily dependent on the downstream Ca^2+^ sensors. In contrast, the actin regulation of [Ca^2+^]_cyt_ via activation/deactivation of Ca^2+^-permeable channels is not very clear at molecular level. Is it the reason of abundance of G-actin, or the ratio of G-actin/F-actin in cytosol? In human, gelsolin is important for actin regulation of Ca^2+^ channels [166,167]. In plants, gelsolin, myosin XI, and the villin/gelsolin/fragmin superfamily proteins also exist, and they are important for actin dynamics [39,74,154,158,161,163,168,169], but their function in regulation of Ca^2+^ channel activation and [Ca^2+^]_cyt_ generation still needs further investigation.
